# Transcriptomic comparison of communally reared wild, domesticated and hybrid Atlantic salmon fry under stress and control conditions

**DOI:** 10.1186/s12863-020-00858-y

**Published:** 2020-05-29

**Authors:** Beatrix Bicskei, John B. Taggart, James E. Bron, Kevin A. Glover

**Affiliations:** 1 Institute of Aquaculture, School of Natural Sciences, University of Stirling, Stirling, FK9 4LA UK; 2 Institute of Marine Research, Bergen, Norway; 3 Department of Biology, University of Bergen, Bergen, Norway

**Keywords:** Atlantic salmon, *Salmo salar*, Domestication, Transcriptome, Microarray, Stress, Farmed escapee, Genetic interaction

## Abstract

**Background:**

Domestication is the process by which organisms become adapted to the human-controlled environment. Since the selection pressures that act upon cultured and natural populations differ, adaptations that favour life in the domesticated environment are unlikely to be advantageous in the wild. Elucidation of the differences between wild and domesticated Atlantic salmon may provide insights into some of the genomic changes occurring during domestication, and, help to predict the evolutionary consequences of farmed salmon escapees interbreeding with wild conspecifics. In this study the transcriptome of the offspring of wild and domesticated Atlantic salmon were compared using a common-garden experiment under standard hatchery conditions and in response to an applied crowding stressor.

**Results:**

Transcriptomic differences between wild and domesticated crosses were largely consistent between the control and stress conditions, and included down-regulation of *environmental information processing*, *immune* and *nervous system* pathways and up-regulation of *genetic information processing*, *carbohydrate metabolism, lipid metabolism* and *digestive* and *endocrine system* pathways in the domesticated fish relative to their wild counterparts, likely reflective of different selection pressures acting in wild and cultured populations. Many stress responsive functions were also shared between crosses and included down-regulation of *cellular processes* and *genetic information processing* and up-regulation of some metabolic pathways, *lipid* and *energy* in particular. The latter may be indicative of mobilization and reallocation of energy resources in response to stress. However, functional analysis indicated that a number of pathways behave differently between domesticated and wild salmon in response to stress. Reciprocal F1 hybrids permitted investigation of inheritance patterns that govern transcriptomic differences between these genetically divergent crosses. Additivity and maternal dominance accounted for approximately 42 and 25% of all differences under control conditions for both hybrids respectively. However, the inheritance of genes differentially expressed between crosses under stress was less consistent between reciprocal hybrids, potentially reflecting maternal environmental effects.

**Conclusion:**

We conclude that there are transcriptomic differences between the domesticated and wild salmon strains studied here, reflecting the different selection pressures operating on them. Our results indicate that stress may affect certain biological functions differently in wild, domesticated and hybrid crosses and these should be further investigated.

## Background

Domestication is possible because some organisms can adapt to the human-controlled environment. The highly contrasting environments that wild and domesticated animals experience thus exert different selection pressures which may in turn promote habitat-specific adaptations [1, 2]. Domestication is beneficial to humans, and advances are achieved via both deliberate directional selection for desired traits, and through inadvertent selection for traits that improve productivity in the culture environment.

In the case of the Atlantic salmon, *Salmo salar* L., economically important production traits including increased growth, late maturation, greater disease resistance and improved flesh quality have been selected for up to approximately 15 generations [3, 4], and this species is now regarded as one of the most domesticated finfish species globally [5]. Simultaneously, unintentional selection to the domestic environment will have occurred through relaxed natural selection and co-selection of traits via genetic linkage and adaptation to the human-controlled environment. For example, predator-avoidance behaviour, that is essential to survival in the wild but insignificant in aquaculture, has changed during domestication of Atlantic salmon even though it has not been selected for [6–9]. Also, reduced survival of offspring of domesticated salmon has been demonstrated in the natural environment [10–13]. This is of concern given that introgression of domesticated salmon escapees in wild populations has been demonstrated in several regions where aquaculture and wild populations co-exist [14–16].

Because the fitness consequences of genetically controlled traits shift during domestication, the optimal investment of resources differs between farm and wild niches. According to resource-allocation theory, since resources available for a given individual are limited, the increased energy demands of one trait may have to be counterbalanced by reducing energy allocation to other, at least momentarily, less important traits. For example, growth is often under strong directional selection in domesticated populations, including Atlantic salmon [17–21]. At the same time, immune function is both necessary and highly energy demanding. Therefore, there is a possible trade-off between growth and immune function as has been proposed for domesticated animals in general [22].

Due to the protected environment of captivity and reliance upon humans to meet key needs, reduced environmental awareness is likely to be a consequence of domestication. This may occur through the decline of information acquisition and transmission systems, such as sensory organs and synaptic activity. Environmental awareness is an evolutionarily highly important trait in the wild, but its reduction is likely to be beneficial for domesticated species in culture through reduction of stress [23]. The effect of domestication on complex traits can be difficult to disentangle such that the activity of traits with multiple biological functions may be enhanced in one species, due to a certain beneficial function, but decreased in another, due to a different function that bears more weight for that organism. As a result, and in contrast to the hypothesized benefit of reduced synaptic activity in domesticated animals, enhanced excitatory synaptic plasticity and its contribution through enhanced memory and learning to effective interaction with humans has been proposed in dogs (*Canis familiaris*) [24].

Response to stimuli, including stress, is context-dependent and among other factors it is influenced by variability in individuals’ experience of the stimulus [25]. Wild and domesticated Atlantic salmon are adapted to different rearing environments and in addition to a wide range of traits [26], their stress responsiveness also differs [19]. Because stress disturbs homeostasis and its restoration is energy demanding, increased stress-responsiveness requires an increased allocation of available resources. To cover this demand, energy is generally directed away from functions that are non-vital and have high energetic costs associated with them, such as growth and reproduction [25], necessitating a further trade-off under culture conditions.

Alteration of gene expression may provide a rapid and plastic response to stress [27, 28]. In addition, since changes in gene expression profiles over time may reflect evolutionary change [29], the study of gene expression is suitable for studying the process of domestication. Given that the Atlantic salmon has now undergone ~ 12–15 generations of domestication selection, resulting in a wide range of genetic-based differences to wild salmon [26], this species represents a good model in which to investigate domesticated-driven changes in the transcriptome. Several previous studies have already utilised the key-attributes of this species [30–32], revealing domestication-driven changes that are likely to be life-stage dependent [33, 34].

The aim of the present study was to i) investigate transcriptomic patterns of wild, F1 hybrid and domesticated Atlantic salmon fry under control and acute stress conditions, ii) identify any existing strain-specific transcriptomic stress responses resulting from gene × family interactions and iii) determine the mode of heritability of the genes identified as differentially expressed between the three genetic groups under both control and stress conditions.

## Results

### Expression data overview

**Fig. 1:**
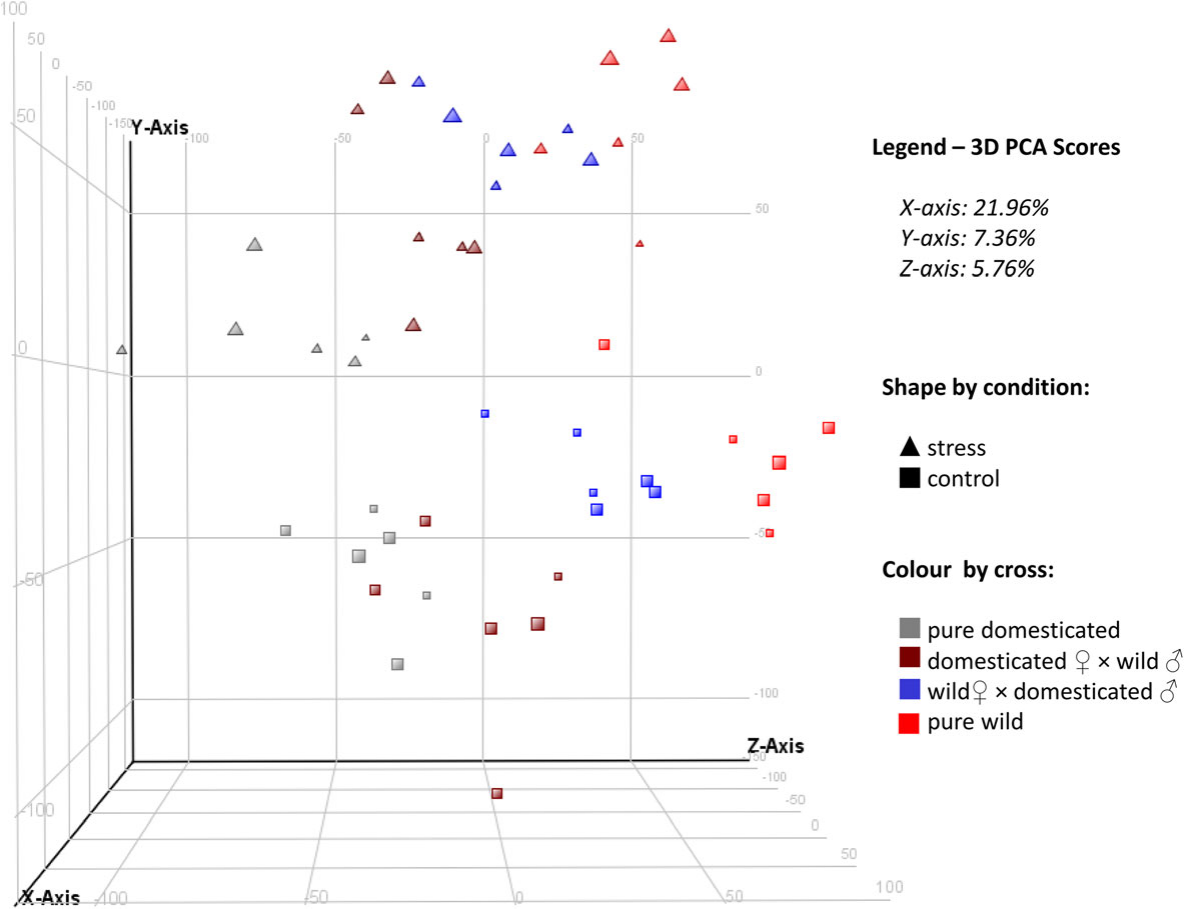
A 3-D representation of the PCA performed on all transcripts that passed quality filtering. Samples are colour and shape coded by the experimental factors. Note the clear distinction between stress and control samples and the general tendency for clustering of samples by state. PCA was conducted on normalised data

3D-PCA clustered the samples according to condition (stress / control) and genetic group (wild / reciprocal hybrids / domesticated) (Fig. 1). Pure wild and pure domesticated groups were found to be the most divergent, whereas reciprocal hybrids tended to be intermediate. Significantly however, the positioning of the reciprocal hybrid groups were indicative of their maternal origin, such that wild dam hybrids tended to be closer to pure wild group, while hybrids of domesticated dams clustered towards pure domesticated group (Fig. 1).

**Fig. 2:**
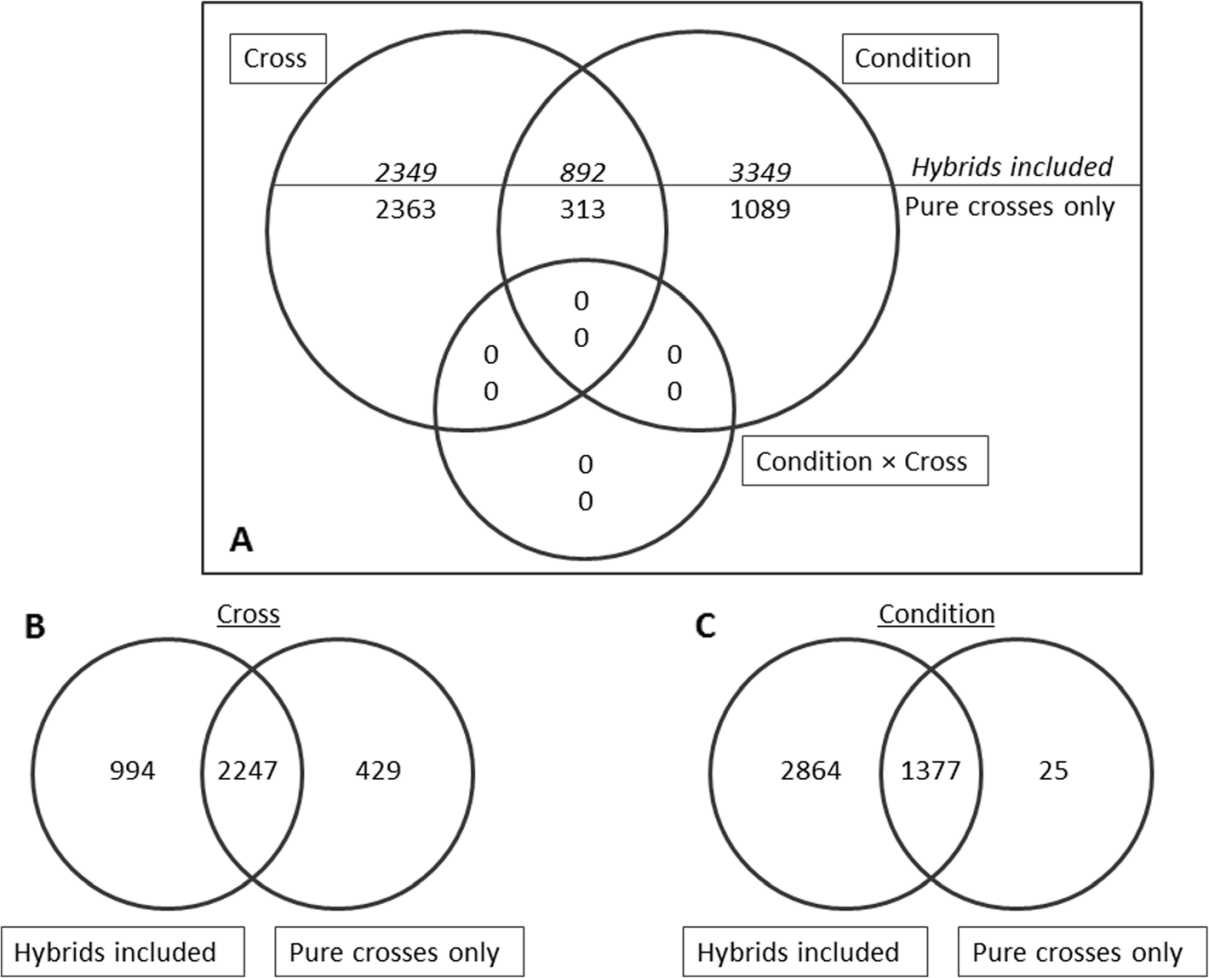
A representation of the number of differentially expressed transcripts based on a 2-way ANOVA. **a**. Transcriptomic differences arising through variation between all crosses (WxW, WxD, DxW, DxD) conditions (stress and control) and the interaction of these two factors. The top numbers reflect statistics for all crosses including the hybrids, whereas the bottom numbers were generated by limiting the 2-way ANOVA to pure crosses only. **b**. The common and unique differences in cross-specific expression with and without consideration of reciprocal hybrids. **c** The common and unique differences arising from exposure to stress vs control conditions and detected with and without consideration of hybrids

Statistical analysis (2-way ANOVA, FDR corrected *p* < 0.05) revealed a number of differentially expressed transcripts among genetic groups and conditions, but no interaction between these two factors exceeded the statistical threshold (Fig. 2a). Separate analyses were performed i) comparing pure wild and domesticated groups only, and ii) considering all four genetic groups i.e. including reciprocal hybrids. Looking at the differential expression explained by genetic group (Fig. 2b), the majority of transcripts (2247) were common to both analyses. In contrast, despite 1377 differentially expressed transcripts being common to both analyses for the factor condition, inclusion of hybrids provided a substantial addition of 2864 unique transcripts (Fig. 2c).

### Functional analysis

Functional analyses of the transcriptomic differences between domesticated and wild strains, as well as in response to stress were performed using two different software packages. Results are presented in Tables 1, 2 and 3.

**Table 1:** Pathways found to be differentially expressed between wild and domesticated stocks under control and stress conditions by both gage and romer packages. The direction of change shown describes the expression of the pathway in the domesticated fish relative to wild counterparts. The terms “2D” and “Mixed” are used to describe pathways in which genes showed bidirectional change. “Genes” refers to the number of genes included in the gene set test

KEGG group	KEGG sub-group	Pathway	Genes	Control		Stress	
				gage	romer	gage	romer
Cellular Processes	Cell communication	Focal adhesion	98	2D	Down	2D	Down
		Gap junction	39			2D	Down
	Cell growth and death	Cell cycle – yeast	54	Up	Up	Up	Up
	Transport and catabolism	Phagosome	76	Down	Down/Mixed	Down	Down/Mixed
		Endocytosis	105	Down/2D	Down	Down/2D	Down
		Peroxisome	54	Up	Up	Up	Up
Environmental Information Processing	Membrane transport	ABC transporters	27			Up	Mixed
	Signal transduction	MAPK signaling pathway	110	2D	Down	2D	Down
		NF-kappa B signaling pathway	64	2D	Down		
		Jak-STAT signaling pathway	56	2D	Down		
		Calcium signaling pathway	72	2D	Down	2D	Down
		PI3K-Akt signaling pathway	149	2D	Down/Mixed	Down/2D	Down
		VEGF signaling pathway	28			2D	Down
	Signaling molecules and interaction	Cell adhesion molecules (CAMs)	64	2D	Down/Mixed	2D	Down
		Neuroactive ligand-receptor interaction	112	Down/2D	Down		
		Cytokine-cytokine receptor interaction	94	Down/2D	Down	2D	Down
Genetic Information Processing	Replication and repair	DNA replication	33			Up	Up
	Transcription	RNA polymerase	27	Up	Up	Up	Up
		Spliceosome	109			Up	Up
	Translation	Ribosome biogenesis in eukaryotes	64	Up	Up	Up	Up/Mixed
		RNA transport	111	Up	Up	Up	Up/Mixed
		Ribosome	118	Up	Up/Mixed	Up	Up/Mixed
Metabolism	Amino acid metabolism	Arginine and proline metabolism	36	Up	Up		
	Carbohydrate metabolism	Amino sugar & nucleotide sugar metabolism	35	Up	Up/Mixed	Up	Up/Mixed
		Galactose metabolism	16	Up	Up/Mixed	Up	Up/Mixed
		Fructose and mannose metabolism	19	Up	Up/Mixed	Up	Up/Mixed
		Glycolysis / Gluconeogenesis	30	Up	Up/Mixed		
	Lipid metabolism	Sphingolipid metabolism	21	Up	Up	Up	Up
		Biosynthesis of unsaturated fatty acids	15	Up	Up/Mixed	Up	Up/Mixed
		Glycerolipid metabolism	25	Up	Up/Mixed		
		Primary bile acid biosynthesis	12	Up	Up/Mixed	Up	Up/Mixed
	Nucleotide metabolism	Pyrimidine metabolism	73	Up	Up/Mixed	Up	Up
	Xenobiotics biodegradation and metabolism	Drug metabolism - cytochrome P450	11	Down	Down/Mixed	Down/2D	Down/Mixed
Organismal Systems	Circulatory system	Vascular smooth muscle contraction	55	2D	Down	Down/2D	Down
	Development	Osteoclast differentiation	69	2D	Down	2D	Down
		Axon guidance	52	Down/2D	Down		
	Digestive system	Protein digestion and absorption	40	2D	Mixed		
		Vitamin digestion and absorption	17	2D	Up/Mixed		
		Mineral absorption	25	Up	Up/Mixed		
		Fat digestion and absorption	19	Up	Up/Mixed	Up	Up/Mixed
		Bile secretion	39	Up/2D	Up/Mixed	Up/2D	Up
		Salivary secretion	32			Down/2D	Down
	Endocrine system	Ovarian steroidogenesis	22	2D	Down	2D	Down
		Thyroid hormone synthesis	33	2D	Up/Mixed		
		PPAR signaling pathway	42	Up	Up		
		Insulin secretion	37			2D	Down
	Environmental adaptation	Circadian entrainment	45	2D	Down	Down/2D	Down
	Immune system	T cell receptor signaling pathway	61	2D	Down		
		Fc epsilon RI signaling pathway	29	2D	Down	2D	Down/Mixed
		B cell receptor signaling pathway	39	2D	Down	Down/2D	Down
		Complement and coagulation cascades	56	Down	Down/Mixed	Down	Down/Mixed
		Hematopoietic cell lineage	38	Down/2D	Down/Mixed	Down	Down
		Chemokine signaling pathway	88	Down/2D	Down/Mixed	Down/2D	Down/Mixed
		Natural killer cell mediated cytotoxicity	40	Down/2D	Down/Mixed	Down/2D	Down
	Nervous system	Glutamatergic synapse	50	2D	Down	Down/2D	Down
		Synaptic vesicle cycle	35	Down	Down		
		Serotonergic synapse	49	Down	Down	Down	Down
		Retrograde endocannabinoid signaling	46			2D	Down
	Sensory system	Phototransduction	14	Down	Down

**Table 2:** Pathways found to be differentially expressed between control and stress conditions in pure wild and domesticated stocks by both gage and romer packages. The direction of change shown describes the expression of the pathway in the stressed fish relative to the control state. The terms “2D” and “Mixed” are used to describe pathways in which genes showed bidirectional change. “Genes” refers to the number of genes included in the gene set test

KEGG group	KEGG sub-group	Pathway	Genes	Wild		Domesticated	
				gage	romer	gage	romer
Cellular Processes	Cell communication	Gap junction	39			2D	Down
	Cell growth and death	Cell cycle	88	Down	Down/Mixed	Down	Down/Mixed
		Cell cycle – yeast	54	Down	Down/Mixed	Down	Down/Mixed
		Meiosis – yeast	41	Down	Down/Mixed	Down	Down/Mixed
	Transport and catabolism	Endocytosis	105			2D	Up
Environmental Information Processing	Signal transduction	Hippo signaling pathway – fly	29	2D	Down	2D	Down
	Signaling molecules and interaction	Cytokine-cytokine receptor interaction	94			2D	Up
		Neuroactive ligand-receptor interaction	112			Up/2D	Up
Genetic Information Processing	Folding, sorting and degradation	Proteasome	40	Down	Down	Down	Down/Mixed
	Replication and repair	Base excision repair	28	Down	Down/Mixed	Down	Down/Mixed
		DNA replication	33	Down	Down/Mixed	Down	Down/Mixed
		Fanconi anemia pathway	35	Down	Down/Mixed		
		Homologous recombination	20	Down	Down/Mixed	Down	Down
		Mismatch repair	18	Down	Down/Mixed	Down	Down/Mixed
		Nucleotide excision repair	35	Down	Down/Mixed		
	Transcription	Spliceosome	109	Down	Down	Down	Down
	Translation	Ribosome biogenesis in eukaryotes	64	Down	Down/Mixed	Down	Down/Mixed
Metabolism	Carbohydrate metabolism	Glycolysis / Gluconeogenesis	30	Up	Up/Mixed		
	Energy metabolism	Oxidative phosphorylation	105	Up	Up	Up	Up
	Metabolism of cofactors and vitamins	Nicotinate and nicotinamide metabolism	12	Up	Up/Mixed		
	Nucleotide metabolism	Purine metabolism	104			Down	Down/Mixed
		Pyrimidine metabolism	73	Down	Down/Mixed	Down	Down/Mixed
	Lipid metabolism	Fatty acid degradation	24	Up	Up		
Organismal Systems	Circulatory system	Cardiac muscle contraction	41	Up	Up		
	Digestive system	Carbohydrate digestion and absorption	15	Up	Up		
		Fat digestion and absorption	19	Up	Up/Mixed	Up	Up
		Gastric acid secretion	27	2D	Up		
		Mineral absorption	25	Up	Up		
		Protein digestion and absorption	40	Up	Up		
		Vitamin digestion and absorption	17	Up	Up		
	Endocrine system	Adipocytokine signaling pathway	35	Up/2D	Up/Mixed		
		Insulin secretion	37	2D	Up		
		PPAR signaling pathway	42	Up	Up/Mixed		
	Excretory system	Proximal tubule bicarbonate reclamation	11	Up	Up		
	Immune system	B cell receptor signaling pathway	39			2D	Mixed
		Fc epsilon RI signaling pathway	29			2D	Down
		Natural killer cell mediated cytotoxicity	40	2D	Down

**Table 3:** Pathways found to be differentially expressed between control and stress conditions in reciprocal hybrids by both gage and romer packages. The direction of change shown describes the expression of the pathway under stress condition relative to control condition. The terms “2D” and “Mixed” are used to describe pathways in which genes showed bidirectional change. “Genes” refers to the number of genes included in the gene set test

KEGG group	KEGG subgroup	Pathway	Genes	W♀D♂		D♀W♂	
				gage	romer	gage	romer
Cellular Processes	Cell growth and death	Cell cycle	88	Down	Down/Mixed	Down	Down/Mixed
		Cell cycle – yeast	54	Down	Down/Mixed	Down	Down/Mixed
		Meiosis – yeast	41	Down	Down/Mixed	Down	Down/Mixed
Environmental Information Processing	Signal transduction	ErbB signaling pathway	40			2D	Down
		HIF-1 signaling pathway	47			Up	Up
		MAPK signaling pathway	110			2D	Up
	Signaling molecules and interaction	Neuroactive ligand-receptor interaction	112	2D	Up	Up/2D	Up
Genetic Information Processing	Folding, sorting and degradation	Proteasome	40	Down	Down/Mixed	Down	Down/Mixed
	Replication and repair	Base excision repair	28	Down	Down/Mixed	Down	Down/Mixed
		DNA replication	33	Down/2D	Down/Mixed	Down/2D	Down/Mixed
		Homologous recombination	20	Down	Down/Mixed	Down	Down/Mixed
		Mismatch repair	18	Down	Down/Mixed	Down/2D	Down/Mixed
		Nucleotide excision repair	35	Down	Down/Mixed	Down	Down/Mixed
	Transcription	Spliceosome	109	Down	Down/Mixed	Down	Down/Mixed
	Translation	Ribosome biogenesis in eukaryotes	64	Down	Down/Mixed	Down	Down/Mixed
		RNA transport	111	Down	Down		
Metabolism	Carbohydrate metabolism	Citrate cycle (TCA cycle)	22	Up	Up	Up	Up
		Galactose metabolism	16	Up	Up/Mixed		
		Glycolysis / Gluconeogenesis	30	Up	Up/Mixed	Up	Up/Mixed
		Starch and sucrose metabolism	21			Up	Up
	Energy metabolism	Carbon fixation in photosynthetic organisms	15			Up	Up/Mixed
		Oxidative phosphorylation	105	Up	Up	Up	Up
	Glycan biosynthesis and metabolism	Glycosaminoglycan biosynthesis - heparan sulfate / heparin	11	2D	Up		
	Lipid metabolism	Fatty acid degradation	24	Up	Up	Up	Up
		Glycerolipid metabolism	25	Up	Up		
		Glycerophospholipid metabolism	44			Up	Up
	Metabolism of cofactors and vitamins	One carbon pool by folate	13			Down	Down/Mixed
	Nucleotide metabolism	Purine metabolism	104	Down	Down/Mixed	Down	Down/Mixed
		Pyrimidine metabolism	73	Down	Down/Mixed	Down	Down/Mixed
Organismal Systems	Circulatory system	Cardiac muscle contraction	41	Up	Up/Mixed		
		Vascular smooth muscle contraction	55	2D	Up	Up/2D	Up
	Digestive system	Carbohydrate digestion and absorption	15	2D	Up		
		Fat digestion and absorption	19	Up	Up	Up	Up
		Gastric acid secretion	27			Up/2D	Up
		Pancreatic secretion	43			Up	Up
		Protein digestion and absorption	40			Up	Up
		Vitamin digestion and absorption	17			Up	Up
	Endocrine system	Adipocytokine signaling pathway	35	2D	Up	Up/2D	Up
		Insulin secretion	37			Up/2D	Up
		Insulin signaling pathway	56			Up	Up
		PPAR signaling pathway	42	Up	Up/Mixed	Up	Up/Mixed
	Environmental adaptation	Circadian rhythm	19			2D	Up
	Immune system	T cell receptor signaling pathway	61			2D	Down
	Nervous system	GABAergic synapse	38			Up	Up
		Glutamatergic synapse	50			2D	Up
		Long-term potentiation	28			2D	Up
		Retrograde endocannabinoid signaling	46			2D	Up

Differences detected in domesticated origin fish relative to wild origin fish tended to be similar in category and direction under both control and stressed conditions. They included down-regulation of *signal transduction* and *immune and nervous systems,* up regulation of *mRNA translation, carbohydrate metabolism* and *lipid metabolism* and *digestive system* and both up and down regulation of some pathways of the *endocrine system* (Table 1)*.* Some of the differentially expressed biological functions were represented by a smaller number of pathways under stress conditions, the most pronounced being the *digestive system*, as a consequence of *protein and vitamin digestion and absorption* and *mineral absorption pathways* only being significantly different under control conditions.

In contrast, pathways differentially expressed in stress relative to control state for wild and domesticated pure genetic groups were less consistent (Table 2). Common transcriptional responses to stress, applicable to both pure genetic groups, included down-regulation of *cell growth and death* and *DNA replication and repair*. In addition, up-regulated *digestive and endocrine systems* appeared to be characteristic of the wild stress response, whereas up-regulated *signalling molecules and interaction* pathways were only found in domesticated fish.

Pathways differentially expressed between the stress and control states for hybrids showed some variation according to the direction of the hybridisation (Table 3). Pathways that were consistent between both hybrids included down-regulation of *cell growth and death*, *DNA replication and repair* and up-regulation of *carbohydrate* and *lipid metabolism* in response to stress. In addition, up-regulation of *signal transduction* and *nervous system* pathways under stress appeared to be characteristic to D♀W♂ hybrids only. Also, up-regulated *digestive* and *endocrine systems* were represented by a larger number of pathways in this hybrid, than in the W♀D♂ hybrid.

### Heritability

**Fig. 3:**
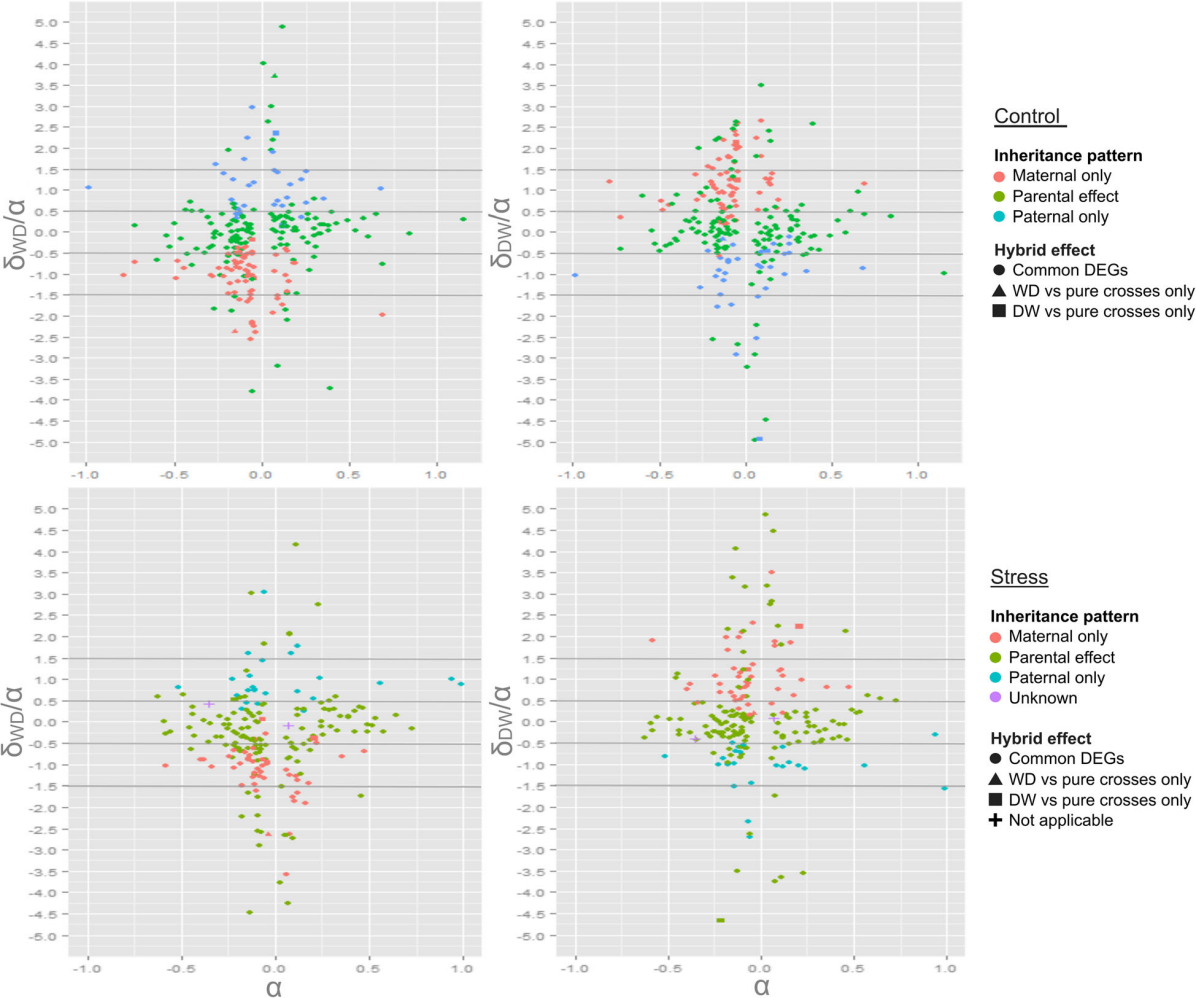
Visual representation of heritability of genes differentially expressed between crosses in control (graphs on top) and stress (graphs on bottom) states. Heritability was plotted for both reciprocal hybrids; W♀ x D♂ (on the left) and D♀ x W♂ (on the right). α > 0 / α < 0 is characteristic of genes that are down/up regulated in domesticated compared to wild fish and − 0.5 < δ/α < 0.5 corresponds to additivity, −1.5 < δ/α < −0.5 to wild dominance, 0.5 < δ/α < 1.5 to domesticated dominance, and if δ/α falls outside the interval − 1.5-1.5, then this suggests over-dominance of the expression of the transcripts studied

**Fig. 4:**
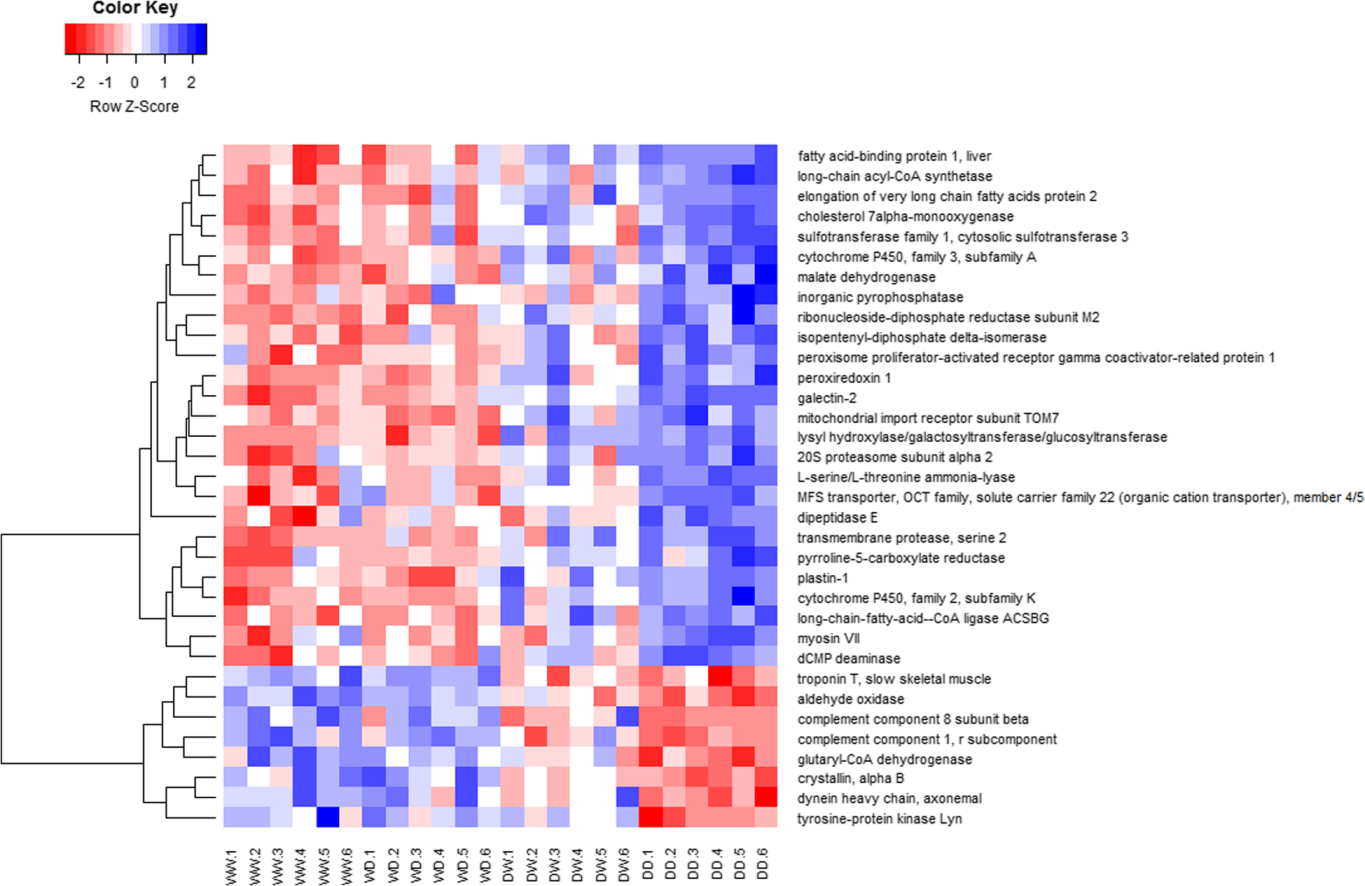
Hierarchical clustering of the normalised expression values of the genes that were identified as wild (over) dominant in the W♀ x D♂ hybrids, and additive/wild dominant in the D♀ x W♂ hybrids under stress conditions. Details of the genes are provided in Additional file 3

Analysis of reciprocal hybrids allowed exploration of gene expression heritability. Additivity (38–46%) accounted for most differential expression patterns detected among the four genetic groups, followed by maternal dominance (18–32%) (Fig. 3, Table 4). On average 42% of the differentially expressed genes exhibited intermediate hybrid expression relative to the pure genetic groups. However, there was a greater difference in the relevance of additivity between the stressed reciprocal hybrids (38 and 46%), than between controls (43 and 41%). The same was true for maternal dominance, with the percentages of differentially expressed genes in the reciprocal hybrids exhibiting this inheritance pattern under the control treatment being relatively consistent (26 and 24%), whereas there was a greater difference between the hybrids under stress (32 and 18%). For most comparisons, maternal dominance was more than double that of paternal dominance. However, in the case of the stressed D♀W♂ hybrids, the difference was considerably smaller; paternal and maternal dominance accounting for 15 and 18% of the differentially expressed genes respectively. There were more pronounced maternal effects detected in W♀D♂ hybrids cf. D♀W♂ hybrids at the expense of additivity under stress conditions, suggesting that the genes responsible for the imbalance are specifically wild maternal and not just maternal dominant. Under stress conditions, genes that were wild dominant in the W♀D♂ hybrids, and were also wild dominant (or additive) in the D♀W♂ hybrids were considerably more abundant than genes that were domesticated dominant in the D♀W♂ hybrids and were also domesticated dominant (or additive) in the W♀D♂ hybrids (34 vs 9 genes). Only seven of these genes were differentially expressed under control conditions, where four of them showed maternal dominance (Additional file 3). The expression of the nominal wild dominant genes (wild (over) dominant in the W♀D♂ hybrids, and wild dominant/additive in the D♀W♂ hybrids) was more consistent in the domesticated crosses than in the wild crosses under stress (Fig. 4). The products of many of the genes were enzymes involved in metabolism, in particular lipid and energy metabolisms.

**Table 4:** Proportions of the differentially expressed genes displaying various inheritance patterns in the reciprocal hybrids relative to the expression of pure crosses under control and stress conditions

Heritability pattern	Control		Stress	
	W♀ x D♂	D♀ x W♂	W♀ x D♂	D♀ x W♂
Wild overdominant	10.7%	8.9%	12.6%	7.6%
Wild dominant	25.8%	11.2%	31.6%	14.8%
Additive	42.9%	40.5%	37.9%	46.2%
Domesticated dominant	11.9%	23.6%	11.2%	17.6%
Domesticated overdominant	8.7%	15.8%	6.8%	13.8%
Number of unique genes	252	259	206	210

## Discussion

Atlantic salmon have been selectively bred since the early 1970s, and as a result, wild and domesticated Atlantic salmon populations now display genetic differences to each other in a wide range of traits [26]. Therefore, Atlantic salmon provides a good model in which to investigate the influence that domestication, including directional selection for economically important traits, has on the transcriptome. Evolutionary responses can manifest themselves in changes in gene expression [29]. In this respect, transcriptional differences between wild and domesticated Atlantic salmon strains have been previously recognised and studied in whole animals [30, 31, 33]. Variation in stress responsiveness between genotypes has previously been reported within commercial Atlantic salmon breeding programs [35]. Considering that wild and domesticated fish are adapted to different environments, some aspects of the stress response might be expected to differ. Hatchery rearing alone [36], as well as longer term domestication have been previously shown to reduce stress responsiveness of Atlantic salmon [18].

In the current study, transcriptional divergence between the domesticated strain and wild population in response to stress was supported by multiple lines of evidence. Separation of stress and control, as well as wild, hybrid and domesticated samples was clearly evident from the PCA analysis. Statistical analysis identified a large number of transcripts differentially expressed between wild and domesticated strains, and in response to stress (stress vs control conditions). Moreover, gene set enrichment analyses found numerous functions that were differentially perturbed between the genetic groups tested and/or in response to stress. It should be noted that using RNA from whole individuals prevented tissue specificity of gene expression being investigated, and this needs to be borne in mind when interpreting the potential biological significance of the data. In addition, although organisms respond to stress via coordinated changes of their gene expression, the response can be further modified through various post-transcriptional controls [27, 28]. These changes will not be detected through comparison of mRNA abundances.

### Effects of domestication on stress response

Domestication involves a combination of selection processes. Traits for desired characteristics are methodically selected for, while additional traits may be inadvertently co-selected. Individuals that respond best to the complete package of selection pressures within the domestic environment are those typically chosen as broodstock to propagate the next generation. Using this approach, gains in population-performance are made from generation to generation. Changes in baseline responses to anthropogenic stimuli have been suggested to be an important aspect of domestication [1, 2]. Increased stress resilience is one of many traits suggested to differentiate wild and domesticated Atlantic salmon [26]. Although differential stress responsiveness might therefore be expected as a signature of domestication, this was not apparent in the study here, which showed no statistically significant interaction between genetic group and stress response in either analysis (+/− hybrid data). Functional analysis, however, suggested that gene expression in some pathways may reflect a strain-specific stress response. Inclusion of hybrid data in ANOVA analyses of transcript expression for fish under stress / control conditions increased the number of differentially expressed transcripts detected, which could be indicative of heightened responsiveness to stress in hybrids.

#### Common responses to stress in fish of wild and domesticated origin

Some cells may respond to stress by reprogramming their metabolism and shifting energy generated by anabolic processes to the repair of stress-induced molecular damage via alteration of the protein translation machinery. In particular, mRNA translation initiation shifts focus from ‘housekeeping’ to repair processes [28]. Overall, stress is thought to reduce global translation throughout the organism in order to preserve cellular energy [27]. This was reflected in the current study with down-regulation of *genetic information processing* in response to stress being detected, including pathways of *replication and repair*, *transcription* and *translation*. *Cell cycle* and *meiosis* pathways, related to *cell growth and death*, were similarly affected. In addition, vertebrate stress response involves increased oxygen uptake and transfer, mobilization of energy substrates and reallocation of energy away from growth and reproduction and towards restoration of homeostasis. Increased metabolic rate, as indicated by positive stress-correlated plasma glucose or oxygen consumption, is also associated with the stress response as is immunosuppression [25, 37]. Data from this study indicated that stress increased metabolic processes, including *carbohydrate, lipid*, and *protein metabolism* and activities involving *co-factors and vitamins*. Up-regulation of *energy metabolism*, *circulatory*, *digestive* and *endocrine systems* and down regulation of *immune pathways* were also characteristic for all stressed fish.

#### Strain-specific stress response

In addition to functional differences shared across the four genetic groups in response to stress, the data also provided evidence of genetic-group-specific stress responses. Indeed, functional differences were found between wild and domesticated pure crosses, as well as between the hybrid strains.

In contrast to the ANOVA analysis, functional analyses of responses to stress identified apparent differences between wild and domesticated origin fish for a number of distinct biological functions. Stress only seemed to affect *signaling molecules and interaction* pathways, *cytokine-cytokine and neuroactive ligand-receptor interactions* in domesticated fish, whereas changes in *metabolic pathways*; *glycolysis/gluconeogenesis* and *fatty acid degradation*, and the majority of *digestive and endocrine system* pathways seemed to be characteristic of wild stress response. The expression of all of the unique differences was enhanced in the stressed compared to control fish. Although many of these changes were marginal, being identified by only one or other of the two analytical tools employed (*gage* or *romer*), the stress-associated up-regulation of *mineral absorption* and *protein digestion and absorption* pathways in wild origin fish cf. domesticated origin fish was fully supported by both packages.

Inclusion of the reciprocal hybrids contributed to an approximately 67% increase in detection of differentially expressed transcripts responding to stress. In addition, there were more pathways differentially expressed in response to stress in reciprocal hybrids than in pure genetic groups. This suggests that the stress response of the reciprocal hybrids was more substantial and/or more variable, than that of the pure genetic groups. Radical genetic changes, such as alleles entering from one population to another, may disrupt adaptation. In hybrid fish, disruption of adaptation may therefore have engendered a need for more extensive responses to stress in order to maintain homeostatic balance. Enriched pathways observed in both hybrids included *signal transduction* and *nervous system*, which were also highlighted in previous studies of fish of wild and domesticated origins [33, 34]. Members of these enriched pathways included *MAPK signaling*, *glutamatergic synapse*, *long-term potentiation* and *retrograde endocannabinoid signaling* all of which are known to be affected by stress and have been implicated in food intake regulation/growth and/or domestication. MAPK is involved in stress response, growth [38] and domestication [34, 39–42], *glutamatergic synapse* has been implicated in stress response, feed intake regulation and domestication [24, 34, 43], *long-term potentiation* has been associated with learning, memory consolidation [44] and domestication [24, 34, 43]. *Retrograde endocannabinoid signaling* is affected by stress [45] and regulates feeding behaviour [46].

Hybrid type varied in some aspects of their response to stress. Overall, there were more differentially expressed pathways detected in D♀W♂ hybrids, than in W♀D♂ hybrids, primarily affecting functional groups of *signal transduction*, *digestive, endocrine* and *nervous system* pathways. These were mainly up regulated in response to stress. Of these functions, perturbation in *protein digestion and absorption, HIF-1 signalling* and *GABAergic synapse* pathways were consistently present in response to stress in D♀W♂ hybrids but absent in W♀D♂ hybrids. *HIF-1* is a transcription factor that functions as the master regulator of oxygen homeostasis and which is induced in response to reduced oxygen availability and/or by other stimulants, including nitric oxide and various growth factors [47]. GABA is considered as one of the most abundant neurotransmitters in the vertebrate central nervous system, and is involved in a number of neuroendocrine processes including the modulation of feeding and stress response, as well as the stimulation of neural development and differentiation and reproduction [48].

Some of the stress responsive functional differences that differed between the pure and reciprocal hybrid genetic groups were shared. For example, a larger number of digestive and endocrine systems related pathways were perturbed in response to stress in the wild, than in the domesticated group. The same trend, affecting the same pathways, was observed in the D♀W♂ hybrids compared to W♀D♂ hybrids. Although largely the result of either *gage* or *romer* failing to detect some of these pathways, it indicates that for digestive and endocrine functions, wild pure and D♀W♂ hybrids had a more consistent and/or stronger stress response, than pure domesticated and W♀D♂ hybrids.

#### Biological functions down-regulated in fish of domesticated origin

Cellular signalling functions in homeostasis by controlling cell replication, differentiation and apoptosis and helps to regulate metabolic events. Stimuli for responses include nutritional state, inflammatory signals or alteration of the organism’s physical environment, these being factors likely to differ between natural and artificial niches. Down-regulation of *signalling* pathways in domesticated fish may be indicative of these animals being better adapted to the more consistent farm environment such that they require less sensitivity or capacity to maintain homeostasis.

Reduction of information acquisition and processing systems, including those involving sensory organs and synapses with transmitter substances for information processing, has been proposed to be a consequence of domestication [23]. The current study supports this hypothesis, with both *cell communication* and *nervous system* pathways being found to be down-regulated in fish of domesticated origin compared to wild. Further support comes from previous studies, where for the same stocks, *cell communication* pathways *gap junction* and *focal adhesion* were observed to be differentially expressed between wild and domesticated origin embryos [34] and *nervous system* related pathways *synaptic vesicle cycle* and *serotonergic synapse* were down regulated in the domesticated origin sac fry [33]. *Glutamatergic synapse* was also identified as differentially perturbed/down regulated in domesticated embryo/sac fry respectively [33, 34]. Generally, decreased serotonergic activity is associated with dominance, boldness and aggression [49]; behaviours more prominent in domesticated fish when compared to wild counterparts in the hatchery environment [50]. Glutamate is a major excitatory neurotransmitter that regulates various behaviours and emotions and is involved in learning and memory [51]. Changes in glutamate metabolism are suggested to have occurred during domestication of dogs [24] and pigs (*Sus scrofa domesticus*) [43]. Expression of glutamate receptors seems to affect the neural control of eating behaviours in pigs [43], with their deficiency having been shown to decrease fear and anxiety in mammals and their up regulation having been hypothesised to enhance excitatory synaptic plasticity in dogs [24]. Up regulation of glutamate activity and hence increased fear and anxiety in dogs compared to wolves is contrary to what one might expect in response to domestication. However, the authors argued that its beneficial effects in terms of strengthening the dogs’ learning and memory abilities outweighed the effects of fearfulness since it aids the accurate interpretation of human behaviour.

Another major down-regulated functional group detected in domesticated fish in the current study, and also in domesticated embryos [34] and in sac and feeding fry [33] belonging to the same strains, was *immune system*. In fish, the neuroendocrine and immune systems are interlinked through shared cytokines and neuropeptides [52, 53] and most of the differentially expressed immune pathways identified in the current study were involved in signalling. Since the importance that particular traits have in the wild, shifts during selection for domestication, the energy invested in them similarly has to be optimised to the new environment. In part this must be achieved through the (re) allocation of resources, and such a trade-off has been identified between growth and immune function, especially in livestock selected for increased production traits [22]. In line with the resource allocation theory, data from the current study showed down-regulation of *immune pathways* in domesticated fish and simultaneous up-regulation of *metabolism*, *endocrine* and *digestive systems* and *genetic information processing*. This is consistent with previous studies that have demonstrated significantly increased growth rates in farmed salmon in comparison with their wild counterparts under identical conditions [17–21, 54].

#### Biological functions up-regulated in fish of domesticated origin

Greater consumption and more efficient utilization of fish feed for growth was reported for Atlantic salmon selected for increased growth over five generations compared to wild counterparts [55]. In addition, selection for growth was suggested to be likely to result in individuals with more active endocrine systems [56]. Such differences were evident from the results of the current study, with up-regulation of *metabolism* and in particular of *carbohydrate* and *lipid metabolism* and *digestive* and *endocrine system* pathways in the domesticated compared to wild fish. In addition, *cellular processes*, such as *cell cycle* and *peroxisome* and *genetic information processing*, including *DNA replication*, *mRNA transcription and translation,* indicative of protein production and growth, were also more highly represented in fish of domesticated origin than in wild origin counterparts.

Functional groupings and regulation of the differentially expressed transcripts detected between fish of domesticated and wild origins were largely consistent between control and stress conditions, as shown by the biological pathways identified and their direction of change. Overall, fewer pathways were identified as differentially expressed in the stress state. This could be a result of individual differences in stress response that may have introduced greater variability in the data and thereby reduced the ability to detect consistent differences in transcript expression. However, the adoption of a pooled design in the current study should decrease the effects of individual variation. Differences were observed in *digestive system*; including *protein and vitamin digestion and absorption* and *mineral absorption* pathways. As these pathways were up-regulated in domesticated compared to fish of wild origin and were up-regulated in response to stress only in the wild fish, it is likely that under stress conditions the increased wild expression masked the difference between wild and domesticated fish of these pathways, resulting in a lack of detectable significant difference..

### Heritability of transcriptomic differences

Most transcriptomic differences detected between the four genetic groups were additive, with c. 40% of differentially expressed transcripts exhibiting intermediate expression in hybrids compared to the pure crosses. Additive genetic variation has been suggested to be characteristic of important Atlantic salmon traits, such as survival [12, 13, 57], growth [17–21, 58], and phenology [13]. Moreover, additive inheritance of gene expression is widespread between conspecifics from widely divergent salmonid populations, including wild and domesticated Atlantic salmon [32–34], brook charr [59] and dwarf and normal lake white fish [60].

Parental effects were differentiated from the effects of domestication by investigating the heritability patterns of the reciprocal hybrids. The majority of the genes showing dominance (18–32%) followed the behaviour of the dam in hybrids and therefore it is clear that that dominance was largely a maternal property, irrespective of genetic origin. Fewer genes displayed paternal dominance behaviour (11–15%), an observation also reported for wild and domesticated brook charr, where 40% of the differentially expressed genes exhibited maternal and 5% paternal dominance [59]. Maternal effects are common in salmonids and have been associated with egg and nest quality [61], and egg and alevin size and survival [12, 62–65]. Maternal effects are likely to be influenced by both genetic and environmental sources of variation [66]. The influence of these components on the phenotype are subject to change over time, and a shift from larger maternal environmental effects to larger genetic effects has been shown during the development of Atlantic salmon [67]. Maternal influence tends to decline over time, including that due to transcriptomic differences [68]. This trend was evident for the extent of maternal over-dominance, for the same strains studied here. The number of transcripts governed by over-dominance steadily decreased from approximately 20% in the embryo stage [34], through a mean of 13 to 5% in fry approximately 3 weeks (Table 4) and 5 weeks [33] post first feeding respectively.

The contribution from additivity and maternal dominance, was consistent between reciprocal hybrids of the control state, but less so in the stress state. This was due to the relatively large proportion of genes that were wild dominant in the W♀ x D♂ hybrids, and were additive/wild dominant in D♀ x W♂ hybrids under stress. This suggests that these genes were under wild dominance, as opposed to maternal dominance regardless of the maternal status. Maternal effects can be adaptive or maladaptive depending on whether the maternal environment is reflective of the offspring’s environment. There are a range of factors known to influence environmental maternal effects including maternal diet and stress experiences [61] that likely vary between natural and farm conditions. Since many of the genes indicative of maternal environmental effects are stress responsive and are involved in lipid and energy metabolism, their expression pattern could be affected by differences in the way wild and domesticated fish metabolise feed, experience stress and produce energy in response to it. In the current study, the expression of the affected genes was more consistent in domesticated origin fish than it was in wild origin fish under stress conditions. This may reflect greater variability of expression of these genes in response to stress in the wild population. Reduced genetic variation has been previously reported for fitness related QTLs in response to domestication, possibly due to genetic sweeps [69].

## Conclusions

This study investigated the functional significance and heritability of transcriptomic differences between Atlantic salmon fry of wild and domesticated origin, maintained under standard hatchery and acute stress conditions. Differences observed were discussed in terms of the contrasting selection pressures acting on natural and aquaculture populations. Although a higher number of responsive pathways were detected in wild origin fish in response to stress, many of the affected pathways were common to fish of both wild and domesticated origin. The major stress-responsive functional groups were indicative of mobilisation and re-allocation of energy. Reciprocal hybrids exhibited similar transcriptomic stress responses to pure domesticated and wild origin stocks, however, some functions that were detected to be differentially expressed between wild and domesticated fish were also found between stress and control hybrids. Additivity and maternal dominance were observed to be the most important modes of inheritance for differential transcript expression detected between the stocks.

## Methods

### Biological samples

The domesticated broodstock used in this study originated from the Norwegian Mowi strain. This commercial strain has been maintained in culture for > 10 generations and has been selected for a range of commercially important traits, for example fast growth, reduced early maturation, improved flesh characteristics and disease resistance. In experimental comparison with wild and F1 hybrid populations, this domesticated strain has been previously demonstrated to display several-fold higher growth rates under hatchery conditions [17–20, 70], and lower survival in the wild [12, 13, 69]. Wild adult broodstock originated from the Figgjo River in southwest Norway. Scale samples from these fish were taken to confirm their wild origin [71]. For further details regarding the genetic background of the strains used in this study, the reader is referred elsewhere [18, 33].

After simultaneously stripping the domesticated and wild broodstock, experimental families (wild, two groups of reciprocal hybrids, domesticated) were established on 23rd November 2011 at the Institute of Marine Research’s experimental fish farm in Matre, Norway. These families are here on referred to as the four genetic groups, and each contained three families as follows: wild = W♀W♂, domesticated = D♀D♂, reciprocal hybrids W♀D♂ and D♀W♂. All of the families within each genetic group were full-siblings to each other (i.e., had 6 unique parents), but the reciprocal hybrids were half-siblings compared to their paternal and maternal pure strains. This required a total of 12 broodstock to generate the 12 families distributed among the 4 genetic groups.

Adipose fin samples from the parents and caudal fin samples from the offspring were retained for DNA profiling. Fertilised eggs were reared under standard hatchery conditions in single family incubators at ambient temperature (4.2–8.1 °C). At the eyed egg stage on 2nd February 2012, all 12 families (3 families × 4 genetic groups) were mixed to generate four replicates, each comprising 30 individuals per family (i.e. 4 replicates each with 360 eggs). These four experimental replicates where thereafter reared in four compartments within the same tank. On 28th March 2012 hatched fry from each of the four replicates were transferred into four separate tanks containing heated water (n tanks = 4, 13 °C, 1m^3^, 45 cm water depth) immediately prior to initiation of exogenous feeding. Thereafter, fry were fed on standard hatchery diet 24 h a day by automatic feeders.

**Fig. 5:**
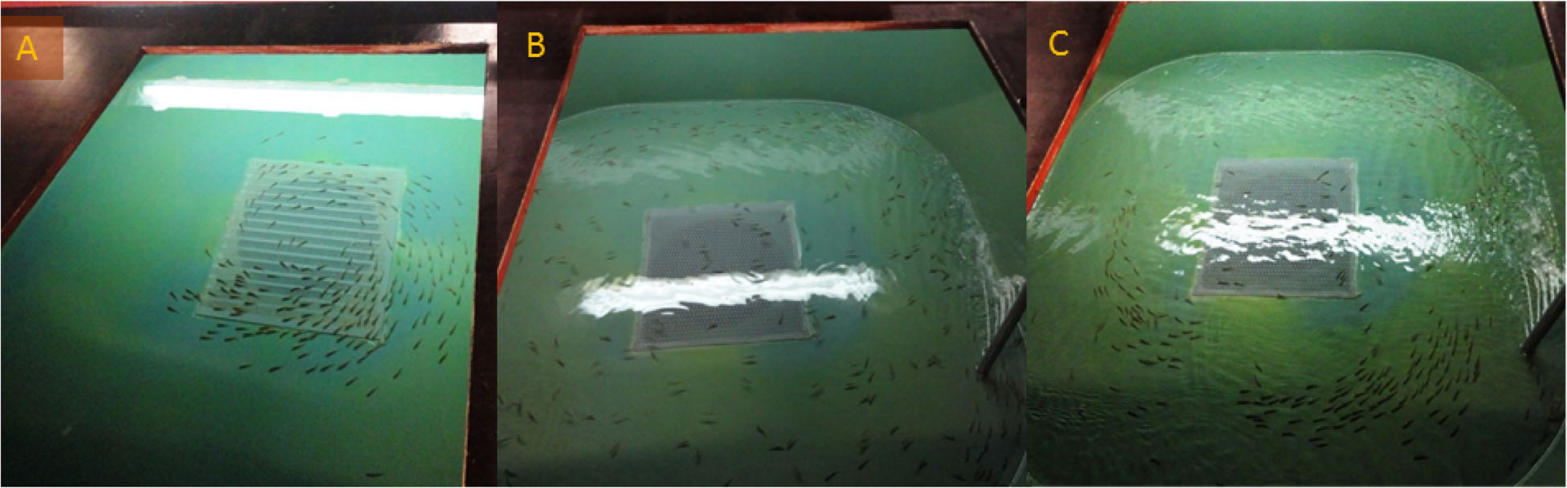
Behaviour patterns of the fish during the experiment. **a**. Prior to stress, fish exhibited schooling behaviour and responded to human presence. **b**. Following the reduction of the water depth, fish broke schooling structure, were distributed randomly in the tanks and did not respond to human presence. **c**. After spending approximately 20 min in shallow water, more structured swimming was observed and fish became responsive to human presence

A stress challenge (including controls) was started on 17th April 2012 (3 weeks post initiation of first feeding, and 985°d post-fertilization). During the stress challenge feeding in all four tanks was stopped. Water level in two of the replicate tanks (the stress replicates) was altered over a 24 h period; 3 h at low depth (2.5 cm) followed by 3 h at normal depth (45 cm). This procedure was repeated 4 times during the 24 h period. In addition to crowding, at low water level, the fish experienced increased water splashing from the inlet feed and increased current velocities. As a first response fish broke schooling structure and were distributed randomly in the tanks (Fig. 5). After approximately 20 min more structured swimming was observed and fish became responsive to human presence, which was not the case in the initial phase (Fig. 5). Water levels in the remaining two ‘control’ tanks were not manipulated. After 24 h, fish from all four tanks were euthanized with metacaine (Finquel® Vet, Scanvacc, Årnes, Norway), and transferred immediately into an RNA stabilisation buffer (3.6 M ammonium sulphate, 18 mM Sodium Citrate, 15 mM EDTA, pH 5.2). After 24 h incubation at 10 °C in this buffer the fry were removed and stored at -70 °C until molecular analysis.

### Family assignment

To assign individual fish sampled from all of the four experimental tanks to their families, and thus genetic group of origin, microsatellite genotyping was performed at the Institute of Marine Research’s molecular genetics laboratory in Bergen, Norway. This laboratory has extensive experience in parentage testing in Atlantic salmon [18, 19, 21], and uses the microsatellite markers implemented here for forensic investigations [72, 73]. A total of 846 fry were genotyped to randomly identify a minimum of 24 individuals from each family and from both conditions (control and stress). As there were two replicates per treatment, this meant that 12 individuals were sampled per family and per control or stress tank. DNA was extracted from tail samples in 96 well plate format using a Qiagen DNeasyW96 Blood & Tissue Kit following manufacturer’s instructions. Five microsatellite loci were amplified in one multiplex PCR; SsaF43 [GenBank: U37494], Ssa197 [GenBank: U43694.1], SSsp3016 [GenBank: AY372820], MHCI [74] and MHCII [75], PCR products were run on an ABI 3730 Genetic Analyser and size-called according to the 500LIZ™ standard. Genotypes were identified using GeneMapper V4.0 (Applied Biosystems, Thermo Fisher Scientific, Waltham, Massachusetts, USA) and family assignment was performed via FAP; Family Assignment Program v3.6 [76]. Individuals unambiguously assigned to families were used in the transcriptomic analysis.

### Microarray experimental design

Although three families per genetic group were included in the stress experiment, scale reading [71] after family-production suggested that one of the wild broodstock used was a farmed escapee. Consequently, in order to not influence the results of the present study, microarray analysis was restricted to include only the two families per genetic group where their origin was identified with confidence.

Microarray analysis was performed using a custom-designed, oligonucleotide microarray platform (Agilent) with four 44 K probe arrays per slide (Salar_3; ArrayExpress accession number A-MEXP-2400). The general design of the microarray has been described in detail elsewhere [77] and further used and validated in a number of subsequent studies [33, 34, 78, 79].

**Fig. 6:**
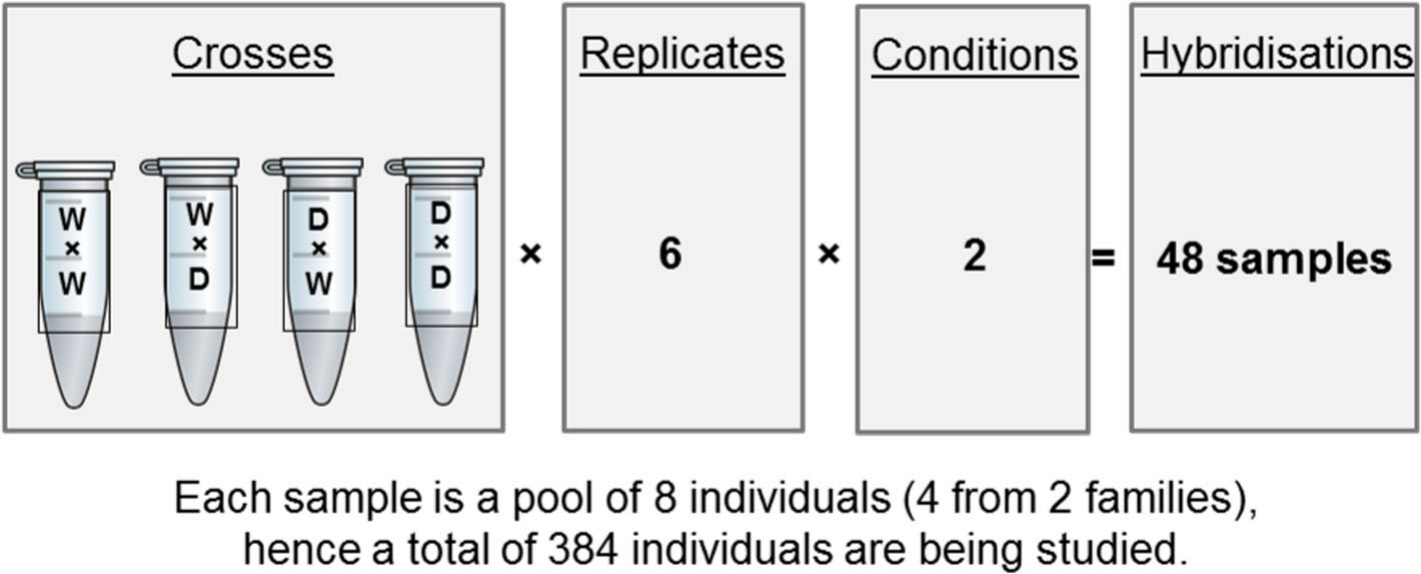
A schematic representation of the experimental design

Dual-label hybridisations were undertaken, with each experimental sample (Cy3 labelled) being competitively hybridised against a pooled reference control (Cy5 labelled) comprising equimolar amounts from each experimental RNA sample. The interrogations comprised 48 separate hybridisations (Fig. 6): 4 genetic groups (wild, domesticated and 2x reciprocal hybrids), 2 conditions (stress and control) and 6 biological replicates (2 tank replicates; 3 pooled samples per tank, each pooled sample comprising 4 individuals from each of 2 families).

### RNA extraction and purification

Whole fry (*n* = 384) were rapidly homogenised in Tri Reagent (Sigma–Aldrich®, St. Louis, U.S.A.) using a Mini-Beadbeater-24 (BioSpec Products Inc., Bartlesville, USA) and RNA extracted following the manufacturer’s instructions. RNA quantity and quality were assessed by spectrophotometry (NanoDrop ND-1000, Thermo Scientific, Wilmington, U.S.A.) and agarose gel electrophoresis respectively. For each biological replicate (hybridisation sample), equal amounts of total RNA from eight individuals per tank were pooled (four fry per family, two families per genetic group) and then re-quantified and quality assessed as described above.

### RNA amplification and labelling

Each pooled RNA sample was amplified (TargetAmp 1-Round Aminoallyl-aRNA Amplification Kit, Epicentre Technologies Corporation, Madison, Wisconsin, USA) according to the manufacturer’s instructions. Following quality control (Nanodrop quantification and agarose gel electrophoresis) each aRNA sample was indirectly labelled and purified. Briefly, Cy dye suspensions (Cy3 and Cy5) in sufficient quantity for all labelling reactions were prepared by adding 42 μL high purity dimethyl sulphoxide (Stratagene, Hogehilweg, The Netherlands) per tube of Cy dye (PA23001 or PA25001; GE HealthCare, Little Chalfont, Bucks, UK). Individual samples (2.5 μg aRNA in 10.5 μL H_2_O) were denatured at 75 °C for 5 min and then 3 μL 0.5 M NaHCO_3_ pH 8.5 and 1.5 μL Cy3 dye added. The reference pool consisted of the same proportions per sample, but 1 μL Cy5 dye was used to label 2.5 μg pooled aRNA. Samples were incubated for an hour at 25 °C in the dark, purified using an Illustra AutoSeq G-50 Dye Terminator Removal Kit (Qiagen GE Healthcare), and concentration, dye incorporation and purity were assessed via spectrophotometer (NanoDrop) with products also visualised on a fluorescent scanner (Typhoon Trio, GE Healthcare).

### Microarray hybridisation and quality filtering

Hybridisation was performed over two consecutive days (24 arrays per day) using the Agilent Gene Expression Hybridisation Kit (Agilent Technologies) as per manufacturer’s instructions. For each reaction, 825 ng Cy5 labelled reference pool and 825 ng Cy3 labelled individual samples were combined in 35 μL nuclease free water and then 20 μL fragmentation master mix added (11 μL of 10x blocking agent, 2 μL 25x fragmentation buffer and 7 μL nuclease free water). The reactions were then incubated at 60 °C in the dark for 30 mins, chilled on ice, and mixed with 57 μL 2x GEx Hybridisation buffer (pre heated to 37 °C), Following centrifugation (18,000 x g for 1 min) the samples were kept on ice until loaded (103 μL) in a structured randomised order onto the microarray slides. Samples from the six biological replicates were divided across different slides, Cy3 fluorescence content (dye incorporation rate x volume) was also taken into consideration. To aid scanning, samples with the most similar amounts of Cy3 were grouped on the same slide. Hybridisation was carried out in a rotating rack oven (Agilent Technologies) at 65 °C, 10 rpm over 17 h.

Following hybridisation, slides were washed in Easy-DipTM slide staining containers (Canemco Inc., Quebec, Canada). First, a 1 min incubation at room temperature (c. 20 °C) in Wash Buffer 1 was performed, with gentle shaking at 150 rpm (Stuart Orbital Incubator). Slides were briefly dipped into Wash Buffer 1 pre-heated to 31 °C, then placed into Wash Buffer 2 (31 °C) for 1 min at 150 rpm. Finally, the slides were transferred to acetonitrile for 10s and then Agilent Stabilization and Drying Solution for 30 s. The slides were then air dried in the dark and scanned within 3 h.

Scanning was carried out at 5 μm resolution on an Axon GenePix Pro scanner at 70% laser power. The “auto PMT” function was enabled to adjust PMT for each channel such that less than 0.1% of features were saturated and so that the mean intensity ratio of Cy3:Cy5 signal was close to one. Agilent Feature Extraction Software (v 9.5) was used to identify features and extract background subtracted raw intensity values that were then transferred to GeneSpring GX (version 13.0) software where the quality filtering and normalisation steps took place. Intensity values ≤1 were adjusted to 1 and a Lowess normalisation undertaken. Stringent quality filtering ensured that features that represented technical controls, saturated probes, probe population outliers or probes which were not significantly different from the background were removed. Agilent feature extraction software was used to determine whether a probe was positive and significant based on a 2-sided t-test, indicating whether the mean signal of a feature was greater than the corresponding background. A probe was retained if it was positive and significant in at least 75% of the arrays in any 4 of the 8 experimental groups. This process resulted in 30,164 of the original 43,413 probes being considered eligible for downstream analysis.

### Microarray data analysis

Three dimensional principal component analysis (3D-PCA) was performed on normalised data in GeneSpring on all transcripts that passed quality filtering. The PCA algorithm also applies a further normalisation step to ‘mean-centre (zero mean) the data. The covariance analysis was computed on the overall gene expression of individual samples. I.e., on all probes that have passed quality filtering. The number of principal components was set to four (default) with the three principal components that explained the major trends of variation shown on the axes. This PCA is solely based on gene expression and independent of experimental grouping.

To investigate genetic group-specific stress response, differentially expressed transcripts were identified in GeneSpring using a 2-way ANOVA. Here, genetic group (wild, reciprocal hybrids, domesticated) and condition (stress and control) were considered as factors and multiple testing correction (Benjamini-Hochberg, *p* < 0.05) was performed. The above statistical analysis was carried out on all four genetic groups, and in addition, on the two pure genetic groups (i.e., wild and domesticated only).

KEGG-based functional analyses of genetic group and condition-specific transcriptomic differences were explored via two analytical approaches, both carried out in *R software v.3.1.3* [80]. First, rank-based *GAGE* analysis (Generally Applicable Gene-set/Pathway Analysis) [81] was performed, implementing Mann Whitney U tests, then the *romer* function from the *limma package* (Linear Models for Microarray Data) [82] was used to achieve more robust results, that are supported by different methods. For *GAGE* results, the software-recommended default FDR “q-value” cut-off < 0.1 was applied. For both techniques, a total of six contrasts were considered. First, to address the primary aim of the experiment, identifying functional differences related to domestication, the domesticated and wild genetic groups were compared under control (genetic group control) and stress (genetic group stress) conditions separately. Then, to identify responses of each genetic group to the stress treatment, stressed fish of wild and domesticated origin were compared to control fish from the corresponding genetic groups (Condition wild and Condition domesticated). Finally, the effect of stress was also investigated for the hybrid groups (Condition WD and Condition DW). To achieve unique KO-probe associations, where multiple probes were assigned to the same KO number, probes with the lowest overall *p*-value based on a 2-way ANOVA were chosen. Since pathways belonging to the human disease functional group are particularly problematic to interpret in fish, this group was excluded from the gene enrichment analysis. The significant pathways jointly supported by both analyses are discussed. The complete lists of pathways identified by the *gage* and *romer* functions are supplied in Additional files 1 and 2 respectively.

To look at heritability of differentially expressed genes between the four genetic groups, 1-way ANOVA (unequal variance) was performed with 5% FDR (Benjamini-Hochberg) and Student Newman-Keuls (SNK) *post-hoc* analysis using GeneSpring. To avoid repeated counting of the same gene, only transcripts that had KEGG annotation available were selected and where multiple probes were present for the same gene, the probe with the highest significance was chosen. The obtained genes were assigned to the following heritability categories:

Maternal effect: differential expression between W♀W♂vs D♀W♂ or D♀D♂vs W♀D♂.

Paternal effect: differential expression between W♀W♂ vs W♀D♂ or D♀D♂ vs D♀W♂.

Parental effect: influenced by both maternal and paternal effects.

Maternal only: unique to maternal effect.

Paternal only: unique to paternal effect.

Additivity (α) and dominance (δ) values were calculated from normalised intensity values (*ni*) for unique differentially expressed genes, where α = (W_ni_ - D_ni_)/2 and δ = ((W_ni_ + D_ni_)/2) - hybrid_ni_. For visualisation α was plotted against δ/α (Fig. 3) using the *ggplot2* package [83]. By definition, a transcript whose expression value in hybrids is midway between that of the parents is additive (perfect additivity: δ/α = 0), whereas a transcript whose hybrid gene expression value resembles one of the two parents more closely is dominant (domesticated dominance, δ/α = 1; wild dominance, δ/α = − 1). By halving the intervals, transcripts were assigned to modes of heritability, as follows:

- additivity if − 0.5 < δ/α < 0.5.

- wild dominance if − 1.5 < δ/α < − 0.5.

- domesticated dominance if 0.5 < δ/α < 1.5.

- over-dominance if δ/α falls out of the interval − 1.5-1.5.

For ease of plot interpretation, genes with |δ/α| > 5 were excluded from the scatter graph but were considered in the heritability table.

Finally, when appropriate, selected annotated (KEGG) gene lists were subjected to hierarchical clustering (Pearson correlation) using the *heatmap.2* function of the *gplots R* package and presented as a heatmap.

## Supplementary information

**Additional file 1.** The complete lists of KEGG pathways identified by the gage function

**Additional file 2.** The complete lists of KEGG pathways identified by the romer function.

**Additional file 3.** Details of the genes that formed the basis of Fig. 6.

## Data Availability

Details of the microarray experiment have been submitted to ArrayExpress (www.ebi.ac.uk/arrayexpress) under accession number E-MTAB-3679 and are therefore openly available. The recording of the microarray experimental metadata complies with Minimum Information About a Microarray Experiment (MIAME) guidelines.
